# Plastic behaviors in hosts promote the emergence of retaliatory parasites

**DOI:** 10.1038/srep04251

**Published:** 2014-03-04

**Authors:** Maria Abou Chakra, Christian Hilbe, Arne Traulsen

**Affiliations:** 1 Evolutionary Theory Group, Max Planck Institute, D-24306 Plön, Germany; 2 Program for Evolutionary Dynamics, Harvard University, Cambridge, MA 02138, USA

## Abstract

Mafia like behavior, where individuals cooperate under the threat of punishment, occurs not only in humans, but is also observed in several animal species. Observations suggest that avian hosts tend to accept a certain degree of parasitism in order to avoid retaliating punishment from the brood parasite. To understand under which conditions it will be beneficial for a host to cooperate, we model the interaction between hosts and parasites as an evolutionary game. In our model, the host's behavior is plastic, and thus, its response depends on the previous interactions with the parasite. We find that such learned behavior in turn is crucial for the evolution of retaliating parasites. The abundance of this kind of mafia behavior oscillates in time and does not settle to an equilibrium. Our results suggest that retaliation is a mechanism for the parasite to evade specialization and to induce acceptance by the host.

Host-parasite coevolution involves constant adaptation and counter adaptation; the host acquires defenses to deflect the costs imposed by the parasite; as a response, the parasite evolves new ways to escape from host defense. Parasites are considered as ‘free-riders' since they exploit the host in order to increase their own reproductive success. For example, brood parasites will lay their eggs in the host's nest and evade all parental care. This host-parasite interaction causes a cascade of co-evolutionary traits such as mimicry, signatures, and timing defenses^1^,^2^,^3^,^4^. However, in spite of their abilities to defend themselves, some hosts are observed to tolerate the parasite. Brood parasites and their hosts are a model system, but despite ample experimental evidence describing this co-evolutionary process, it is still not clear under which conditions the hosts are compelled to accept parasitism^4^,^5^,^6^,^7^,^8^,^9^,^10^.

Cooperating with a parasite has detrimental consequences. Two hypotheses have been proposed to explain when accepting behavior is evolutionarily possible^4^,^10^. The evolutionary lag hypothesis posits that hosts would naturally evolve to reject the parasites. However, there is a lag in their response and insufficient amount of time has passed for the adaptation to occur. The equilibrium hypothesis posits that the costs associated with rejection outweigh acceptance. Rejection is costly, and thus, less adaptive. Both hypotheses have some support, but conclusive evidence is wanting^4^,^5^,^6^,^7^,^8^,^9^,^10^.

Alternatively, Zahavi proposed a ‘mafia hypothesis' which posits that hosts accept because they fear retaliation by the parasite^11^. Empirical evidence shows that both great spotted cuckoos (*Clamator glandarius*) and brown-headed cowbirds (*Molothrus ater*) exhibit such retaliatory behavior. These parasites have different strategies and host types, yet, both were observed to punish their respective hosts (*e.g.* magpies, *Pica pica*, and prothonotary warblers, *Protonotaria citrea*) for rejecting their eggs by destroying entire successive nests. In order to avoid repercussions from the retaliating parasitic behavior, hosts tend to accept a certain degree of parasitism^12^,^13^. The mafia hypothesis works when retaliation is sufficiently costly to the host in comparison to the consequences of accepting parasitism. In particular, this assumes that the host's young are raised alongside of the parasite's young, which is the case in these examples^12^,^13^. However, the behavioral consequences of retaliatory punishment are still unclear. Here, we investigate the emergence of retaliatory behavior and explore the host's response to such ‘mafia-like' behavior in parasites by an analytical model and numerical simulations.

Direct behavioral adaptations and counter-adaptations render the brood host-parasite system described above ideal for theoretical investigations^5^,^14^,^15^,^16^,^17^,^18^. Robert *et al.* showed, by including repeated interactions, that parasite retaliation oscillated temporally; they observed that retaliation evolves when host rejection is abundant^19^. However, typical behavioral models^19^,^20^,^21^ have only considered two types of hosts (rejecters or accepters) and parasites (retaliators or non-retaliators). In these previous models, host do not exhibit any plastic response to parasites. Since hosts are incapable of adapting their behaviors between clutches or after an interaction with a parasite, previous models could not address whether the retaliatory mafia truly influences the decision of the host in subsequent interactions. In contrast, in nature some hosts change their behaviors by learning within a single breeding season^3^,^22^,^23^. Hence, in our model, we allow hosts to react on their past experience with parasites: A host could accept parasitism unconditionally (first clutch) or conditionally (reject in first clutch and accept in second clutch). Spiteful behaviors can emerge, parasites who retaliate, but do not return to the host. Also, opportunistic behaviors can emerge, parasites who return to the host without retaliating. Given all those behaviors interacting together, will ‘mafia-like' behavior evolve? When should a host accept conditionally versus unconditionally? Additionally, how much parasitism should a host accept? To address these questions, we construct a model that captures these more complex host-parasite interactions.

## Results

### The model

We model the interaction between hosts (e.g. magpies, *Pica pica*, and prothonotary warblers, *Protonotaria citrea*) and parasites (e.g. cuckoos, *Clamator glandarius*, and cowbirds, *Molothrus ater*) as an evolutionary game^13^,^24^,^25^. These species tend to interact with each other over several seasons. In each season a parasite can lay more than a single egg in the host's nest without fear that the parasite nestling will evict its siblings out of the nest; both the young of the host and parasite are raised together^1^,^12^,^22^. Thus, in this system there are repeated interactions within a breeding season. We classify the host-parasite interactions into four stages: (i) During the first parasitism stage the parasite lays a certain number of eggs in the nest and the host will choose to accept these eggs or to reject them. (ii) If the host rejects, the parasite could depredate and destroy the host's nest during the first depredation stage. This stage occurs after the host rejects, which could be any time between egg laying and nestling hatching^12^. (iii) The second parasitism occurs only when the parasite returns to the same host to lay additional eggs after rejection or after the host reclutches. (iv) The second depredation stage occurs after a host rejects the returning parasite.

In the model, a parasite's behavior is determined by the number of eggs laid per host nest, whether it is a retaliator (mafia) or not, and whether it returns to the same host or not. Similarly, the host's behavior is determined by the number of parasitic eggs tolerated, whether retaliating parasites are accepted or not upon their return, whether non-retaliating returning parasites are accepted or not, and whether the host ejects the parasitic eggs or abandons the entire nest. A host's behavior can differ between stages. For instance, a host can reject in the first parasitism stage, but choose to accept in the second parasitism stage. These behaviors can become complex quickly, and thus, it is important to distinguish between each response that is possible by a parasite (*e.g.* number of eggs laid per nest, retaliation, returning to the same host) and host (e.g. acceptance, rejection, eggs tolerated, accept retaliators or not). We assume that a host will lay one clutch during the breeding season unless they are depredated on, with a maximum of three clutching attempts in a breeding season^22^,^26^.

This is captured by an asymmetric evolutionary game played between one parasite and multiple hosts selected at random from their respective populations; this setup mimics natural scenarios where parasites, depending on the species, can lay between 20 and 40 eggs per breeding season^1^. Using evolutionary game theory, we analyze host-parasite interaction at each stage. Over time, behaviors evolve based on the fitness^24^. In each season, a parasite has the capacity to lay a certain number of eggs that is allocated among hosts. Non-mafia parasites lay a total of *β_N_* eggs while mafia parasites lay a total of *β_M_* eggs. When *β_M_* > *β_N_* the emergence of mafia behavior is trivial, and will not be discussed herein. Instead, we assume *β_M_* ≤ *β_N_*, that is mafia-behavior may be costly for the parasite (for example, because the parasite may mistakenly eliminate its own offspring, or because of cognitive and energy costs^27^). A host lays *b_h_* eggs in each clutch, and under parasitism incurs a cost *c_p_* if parasitism occurs, *c_n_* if a nestling is raised, and *c_r_* if forced to re-clutch. To calculate the average fitness of a parasite we consider the average fraction of accepted eggs reared by the host, whereas to calculate the average fitness of a host we consider the average number of its eggs successfully reared.

For a concrete analysis we consider two models, a minimalistic version with few strategies that can be analyzed analytically and a more complex version which we analyze with simulations. Both models allow hosts to change their behavior between each interaction (plastic response).

### Minimalistic model

We first consider the case where the parasite lays a single egg per nest, *b_p_* = 1 and only consider three types of host and two types of parasites. Host types are accepters (hosts that accept after first parasitism stage), conditional accepters (hosts that accept only after retaliation), or rejecters (hosts that always reject). Parasite types are non-mafia (non-retaliators that switch to a new host after rejection) or mafia (retaliators that depredate the nest and re-parasitize the same host). The fitness of both, host and parasite, depend on their evolved strategies.

**Figure 1:**
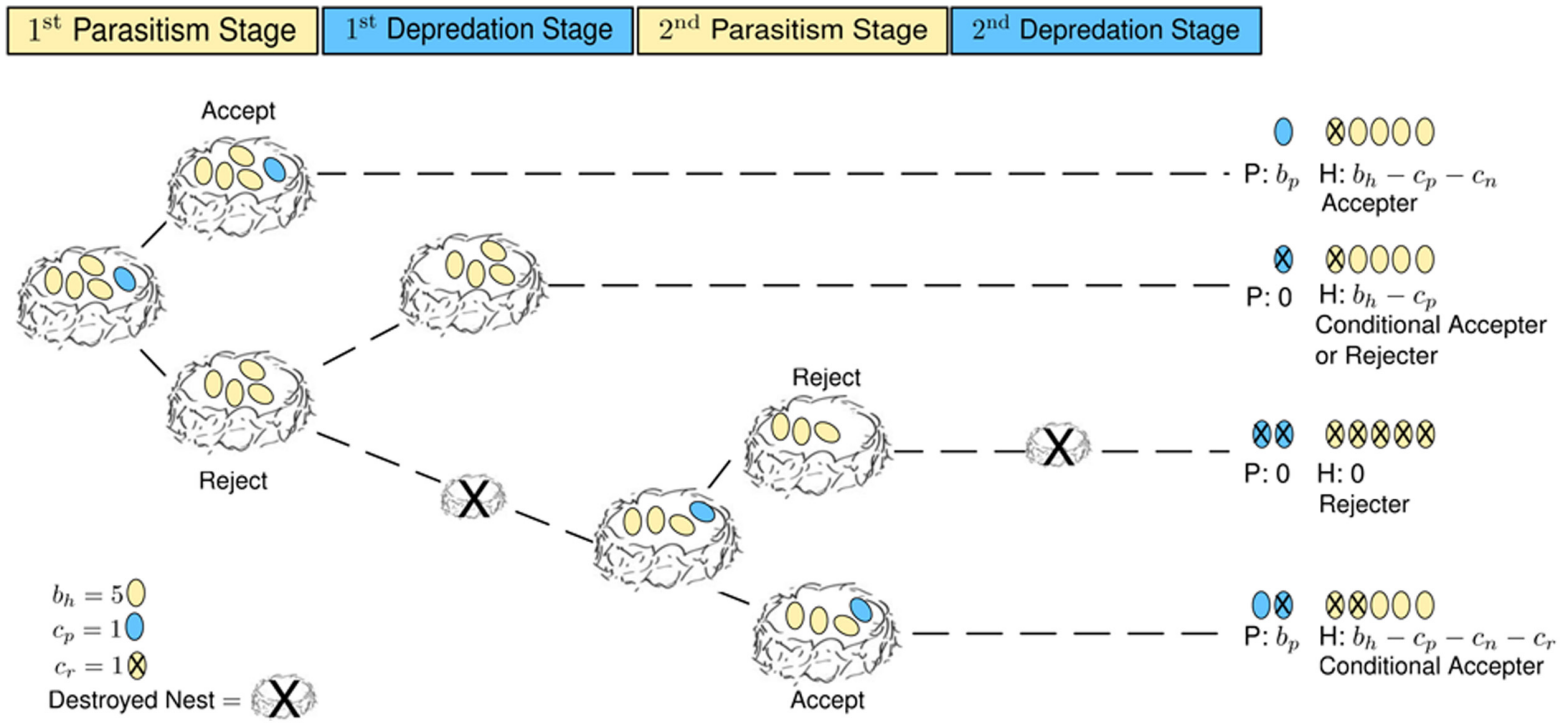
Game tree for the host-parasite interaction in the minimalistic model. The host lays *b_h_* eggs in a clutch which can become parasitized with a single egg. The hosts fitness may be reduced by *c_p_* (when the host is parasitized), by *c_n_* (when the parasite's nestling is raised), and by *c_r_* (when the host is forced to re-clutch). The parasite gains the accepted egg, *b_p_*.

We can infer the average fitness of a host from the game tree in Fig. 1. We suppose that in each breeding season, the host lays *b_h_* eggs in a clutch which can become parasitized with a single egg, *b_p_* = 1. The host may accept the parasitic egg incurring a parasitism cost *c_p_* (parasites often remove or destroy host's eggs, typically as many eggs as they lay in a nest^1^) and a nestling cost, *c_n_* (for raising the parasite's young); the parasite gains the accepted egg, *b_p_*. Alternatively, the host may reject the parasitic egg, but risk retaliation whereby the parasite destroys the whole clutch. In case of non-retaliation, the host just incurs a parasitism cost, without any gain for the parasite. In case of retaliation, the host lays a second clutch at a cost, *c_r_*, which again we assume is parasitized. In this situation, the host may accept the second parasitic egg and incur a parasitism cost, nestling cost and a reclutching cost; the parasite gains the accepted egg, *b_p_*. Alternatively, the host may reject the second parasitic egg, and due to the retaliatory behavior of the parasite, both end up with nothing (we assume retaliators always return and all future clutches are destroyed).

For the analytical model, the three types of hosts, accepters *A*, conditional accepters *C*, and rejecters *R*, have respective frequencies denoted by *y_A_*, *y_C_* and *y_R_* such that *y_A_* + *y_C_* + *y_R_* = 1. Similarly, the two types of parasites, non-mafia *N*, and mafia *M*, have respective frequencies denoted by *x_N_* and *x_M_* such that *x_N_* + *x_M_* = 1.

To calculate the average fitness of a parasite we compute the average acceptance rate for each parasite type. For a non-mafia strategist, since they lay a single egg per nest, the average number of eggs accepted per parasitized host is directly proportional to accepter host frequencies, *y_A_*. For a mafia strategist, on the other hand, the average number of eggs laid per parasitized host is 1*y_A_* + 2*y_C_* + 2*y_R_* = 2 − *y_A_*, whereas the expected number of accepted eggs per parasitized host is *y_A_* + *y_C_*. Thus, the average fitness (ratio of accepted to laid eggs) of a parasite is given by 
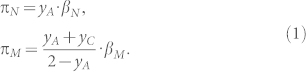
We infer from equations (1) that when all hosts are accepters (*y_A_* = 1) the fitness of non-mafia and mafia-types are *β_N_* and *β_M_*, respectively, such that non-retaliatory parasites have the higher fitness. When, on the other hand, all hosts are conditional accepters (*y_C_* = 1) the non-mafia parasites are always rejected and every second lay by a mafia parasite is accepted. In such a host population, retaliation pays. Finally, when all hosts are rejecters (*y_R_* = 1) both types of parasites fail to reproduce.

To calculate the average fitness of a host, we first calculate the probability with which each parasite type visits the host. A non-mafia parasite needs to visit *β_N_* different hosts (one host for each egg), whereas a mafia parasite requires on average *β_M_*/(2 − *y_A_*) different hosts (which is between *β_M_* if all hosts are accepters, and *β_M_*/2 if not a single host is an accepter and the parasite returns to all of them). Thus, the average fitness of a host is 
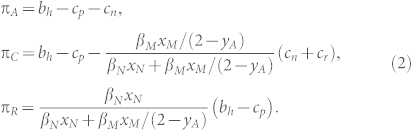
In the absence of retaliatory parasites (i.e., *x_M_* = 0), rejecters and conditional accepters have a higher fitness than accepters. This relation, however, is reversed when retaliation is common (i.e., *x_M_* = 1). Moreover, conditional accepters dominate rejecters (in the sense that *π_C_* ≥ *π_R_*, and *π_C_* > *π_R_* if the parasite population contains any mafia strategists, *x_M_* > 0). Thus, for modeling the evolutionary dynamics, we simplify our model by neglecting unconditional rejecters *R* in the host population in the following.

**Figure 2:**
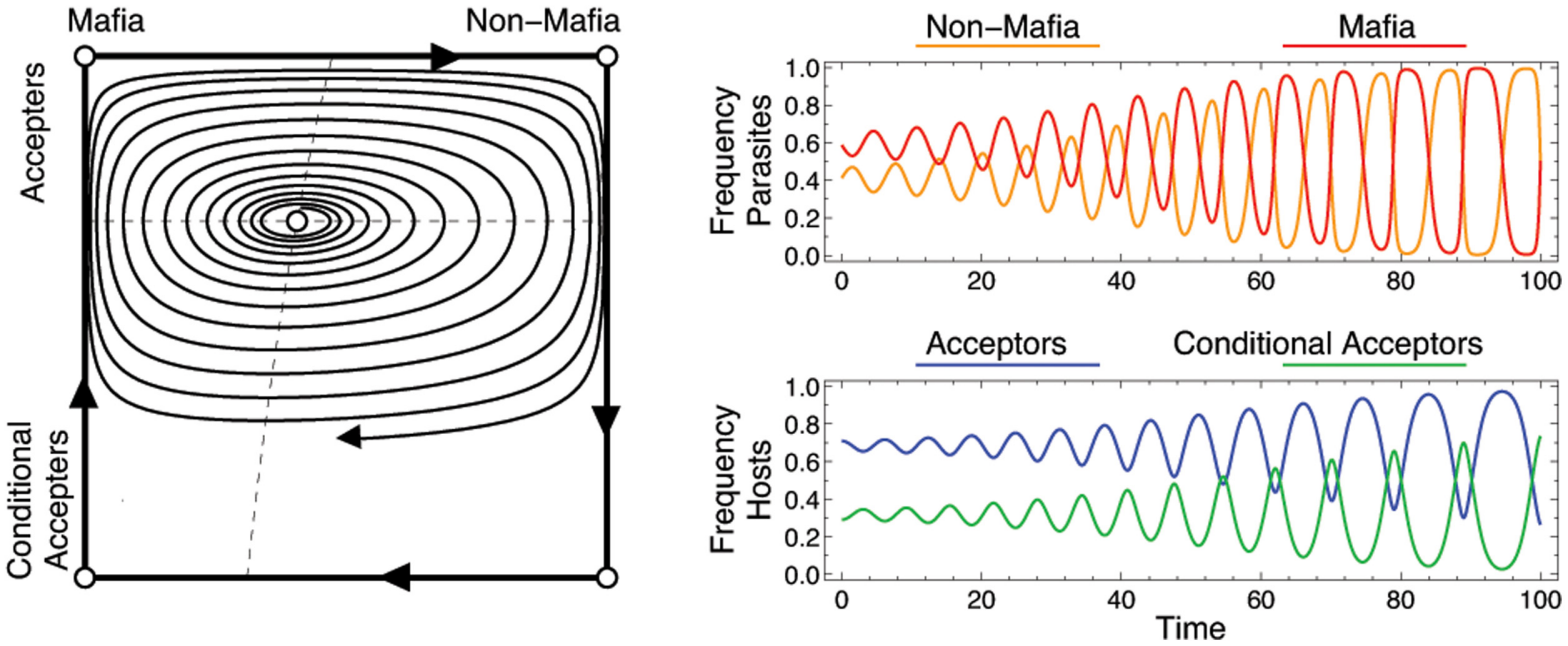
Evolutionary cycles in the replicator dynamics for the host-parasite interaction. The left graph shows the state space, with the horizontal axis corresponding to the parasite frequencies (mafia behavior is frequent at the left side, and rare at the right side), whereas the vertical axis gives the host frequencies (accepters are frequent at the top, and conditional accepters at the bottom). Dashed lines show the isoclines of the system, their intersection gives the unique, but unstable equilibrium in the interior. Even if the initial population is close to the equilibrium, evolutionary orbits follow periodic cycles with increasing amplitude, as shown in the right graph (parameters *b_h_* = 5, *c_r_* = *c_p_* = *c_n_* = 1, *β_M_* = 18 and *β_N_* = 20).

We model the dynamics in this host-parasite population using the replicator dynamics for asymmetric games^24^; that is, we assume that the evolution of strategies can be described with the following two equations: 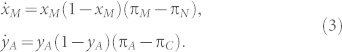
The dynamics of this host-parasite interaction is cyclic (Fig. 2). When the non-mafia parasites are in the majority, conditional accepter hosts have the higher fitness. As the frequency of these conditional accepters increases, it becomes beneficial for the parasites to retaliate against these *C* type hosts, and thus, mafia parasites increase in frequency. As soon as mafia parasites are common, it is optimal for hosts to give in without delay, leading to an increase in accepter hosts. This, in turn, makes it needless for parasites to retaliate, leading the parasite population back to the non-mafia strategy.

All of the pure population states are unstable fixed points. Moreover, the cyclic dynamics has a fixed point (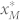, 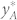) in the interior of the state space, 
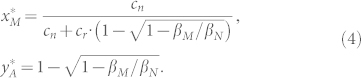
For the parasites, the equilibrium fraction of mafia strategists depends on their relative baseline fitness *β_M_*/*β_N_*, and the costs incurred by the host, the nestling cost *c_n_* and the re-clutching cost *c_r_*. Higher re-clutching costs decrease the equilibrium abundance of mafia parasites. As *c_r_* is increased, it becomes riskier for hosts to reject the parasite, and thus, the mere threat of retaliation is often sufficient. Surprisingly, the equilibrium frequency of mafia parasites increases if their relative baseline fitness *β_M_*/*β_N_* is reduced. This counterintuitive effect of the relative baseline fitness can be understood by analyzing the equilibrium frequency of accepting hosts. A low *β_M_* reduces the probability that a given parasitic egg is laid by a mafia-parent, thereby making hosts more inclined to reject foreign eggs in their first clutch. This in turn promotes the spread of mafia behaviors that enforce the host's acceptance in subsequent clutches. Overall, this effect outweighs the initial disadvantage of a lower baseline fitness *β_M_*.

Notably, the host's equilibrium abundance is independent of the costs *c_p_*, *c_r_* and *c_n_*. An increase of these costs therefore does not affect the eventual equilibrium fraction of accepters and conditional accepters in the population. Instead, changes in theses costs are only reflected by strategy changes of the parasites (this somewhat counterintuitive effect is characteristic for mixed Nash equilibria, see for example^28^).

However, the interior equilibrium is evolutionarily unstable and a numerical analysis suggests that there is a limit cycle (as this cycle is close to the boundary of the system in our case, in a finite population extinction of one type in each population is a likely outcome). These dynamical results deviate from the equilibrium hypothesis: although a cost-benefit equilibrium exists, any small perturbation will drive the population away, and once left, there is no evolutionary force leading back. Interestingly, our model suggests that the resulting evolutionary lag does not diminish over time.

### Complex model

**Figure 3:**
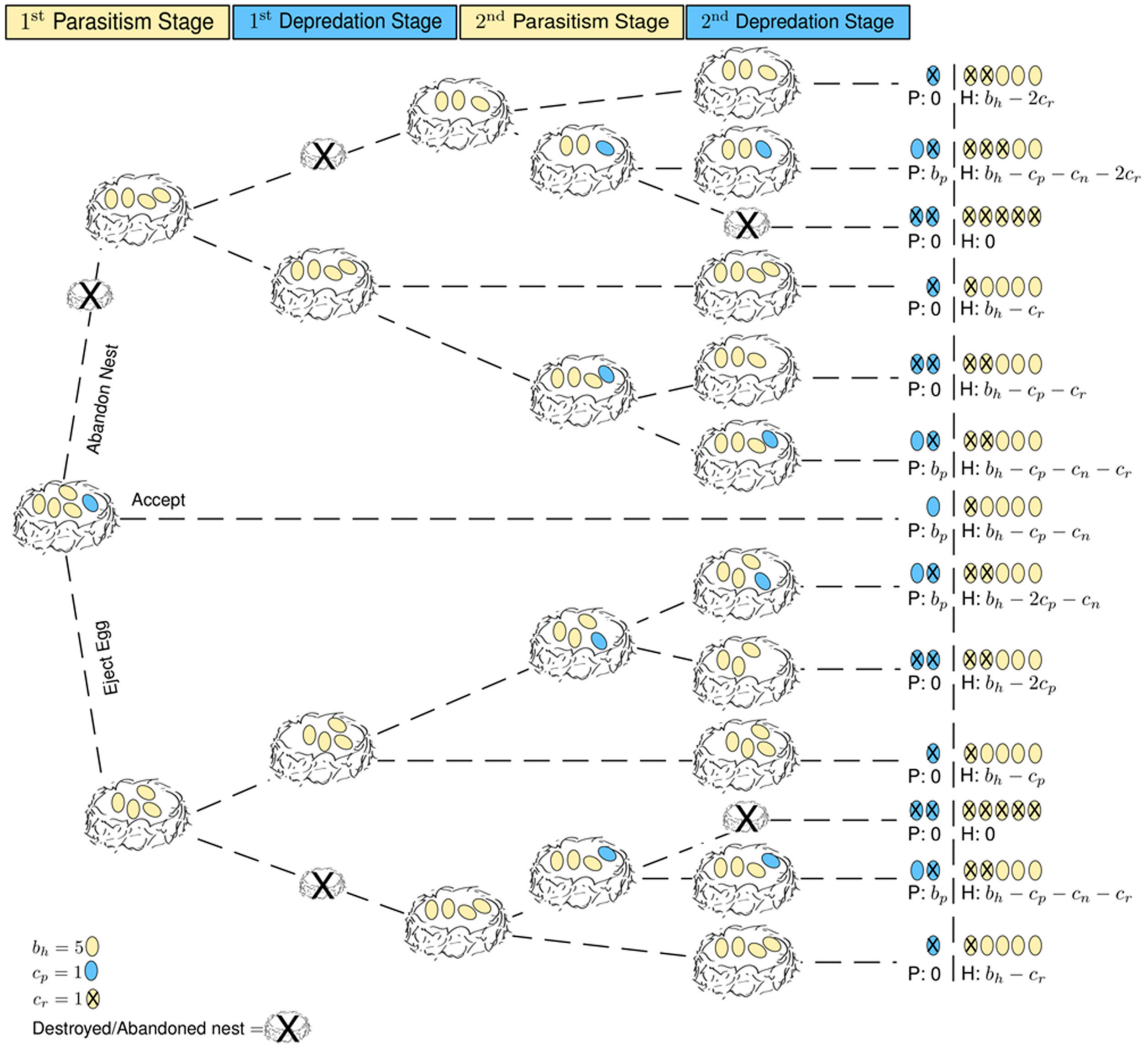
Game tree of host-parasite interaction in the complex model. A host lays *b_h_* eggs in a clutch which can become parasitized by *b_p_* parasitic eggs. Depending on the host's threshold trait *t*, the host may accept the parasitism (if *b_p_* ≤ *t*) incurring a parasitism cost *c_p_*, a nestling cost *c_n_*. When hosts reject parasitism (if *b_p_*>t), they risk being forced to re-clutch, resulting in costs *c_r_*. The parasite gains the accepted egg, *b_p_*.

The aforementioned results give a proof of principle; they show how mafia behavior can emerge in a comparably simple setup. But how many eggs should the parasite lay and the host accept? For this, we consider a more complex system (Fig. 3) where the parasite can lay more than a single egg into a nest and where the host can determine a threshold for the number of parasitic eggs that will be accepted. Moreover, the host can now choose between abandoning the nest or ejecting the parasitic egg, which is typical of the considered host species (magpies or prothonotary warblers)^1^.

**Figure 4:**
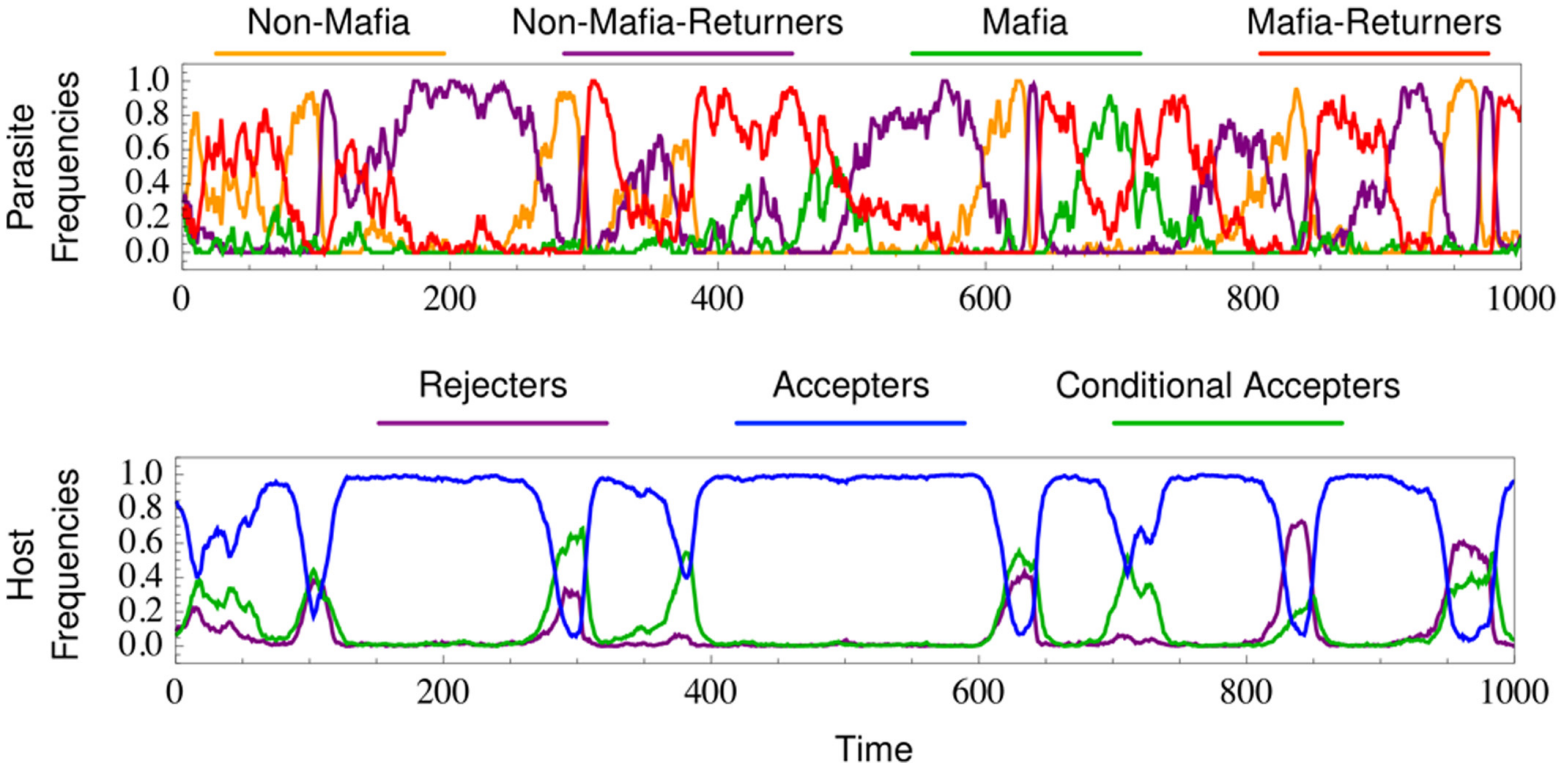
Simulation of the complex model of host-parasite interactions depicted in Fig. 3. The dynamics are reminiscent of cyclic pattern between mafia and non-mafia parasite and among rejecter, conditional accepter, and accepter hosts. Parameters, *G* = 1000, *N_p_* = 50, *N_h_* = 1000, *b_h_* = 5, *β_N_* = 20, *β_M_* = 18, *μ_p_* = 0.005, *μ_h_* = 0.01, *c_n_* = 1, *c_r_* = 1, *c_p_* = 1.

We analyse the host-parasite dynamics using large-scale individual based simulations, cf. Methods section for more details. We observe dynamics reminiscent of cyclic patterns between mafia and non-mafia parasites and among rejecter, conditional accepter, and accepter hosts, Fig. 4. Mafia parasites are most prevalent when acceptance is conditioned upon retaliation and this slowly decreases as the host population changes from conditional-accepters to just accepters until non-mafia parasites increase in frequency. We also observe that almost all mafia parasites return to their hosts. Opportunistic or spiteful behaviors emerge only at low frequencies, Fig. 4. Opportunistic parasites do not punish, they just return to the host, while spiteful parasites punish but do not return. These behaviors take advantage of the increasing accepter hosts in the population, but tend to be unsuccessful since most conditional hosts only accept returning parasites who destroy their nest. This suggests that all behavioral changes (between the first clutch to the second) require retaliation and a second parasitism from the same parasite.

**Figure 5:**
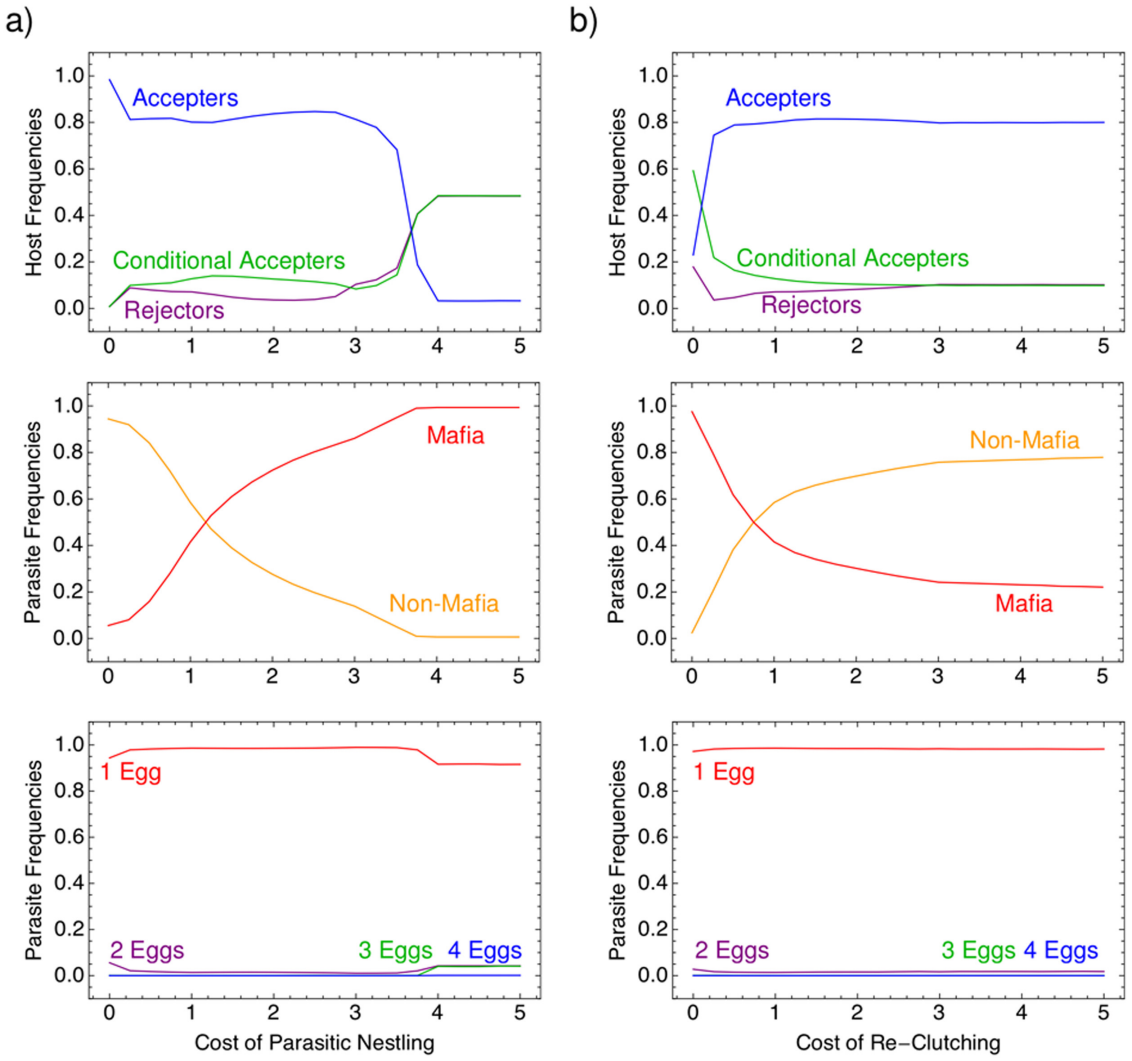
Exploring (a) the cost of parasitic nestling, *c_n_* and (b) the cost of re-clutching, *c_r_* on the relative abundance of strategies in the population. (a) As nestling cost increases the frequency of retaliating parasites increases. (b) As re-clutching cost increases the accepter-hosts increase in frequency, and since there is no need to enforce acceptance, mafia parasites decrease in frequency. Parasites again evolved to lay a single egg for all nestling and re-clutching costs. Parameters, *G* = 1000, *N_p_* = 50, *N_h_* = 1000, *b_h_* = 5, *β_N_* = 20, *β_M_* = 18, *μ_p_* = 0.005, *μ_h_* = 0.01, *c_p_* = 1, *c_r_* = 1 in a) and *c_n_* = 1 in b).

Furthermore, costs imposed on hosts by the parasite drive changes in behavior. In general, nest abandoning occurs when parasites are non-retaliators and re-clutching costs are low. Otherwise the host just ejects the eggs. Simulations show that as nestling cost increases, the frequency of retaliating parasites increases, which is in agreement with Eqs. [4]. Parasites have to enforce acceptance with increasing nestling cost, since hosts prefer to reject when nestling costs are high. Similarly, increasing re-clutching cost increases accepter hosts frequency, and as a result, mafia parasites decrease in frequency, Fig. 5.

Interestingly, the majority of the parasites evolved to lay a single egg per interaction, Fig. 5, which is in line with observed patterns^1^. This was independent of all costs (parasitism, nestling, and re-clutching) with the assumption that there is no extra cost to finding additional nests. Intuitively, since a host that accepts *i* parasitic eggs also accepts any lower number, a parasite that lays one egg per nest will have the highest acceptance rate.

## Discussion

Punishing free-riders or individuals who break group rules is observed both in animal and human societies^29^,^30^,^31^,^32^,^33^. However, punishment by ‘rule breakers' or free-riders seems to be less common and occurs when individuals use violence or threats to compel another to do their bidding. This is most successful when individuals are reputed to punish when others refuse to comply^34^, for example, mafia organizations or school bullies. When the mafia hypothesis was originally proposed by Zahavi in 1979^11^, he was opposed on the grounds that such behavior is costly for the parasites, without having direct benefits^27^: mafia behavior would reduce the fitness of rejecting hosts, but the parasite's ejected chick would have nothing to gain from the parent's action. However, herein, we have shown that retaliatory behavior emerges when hosts have plastic behaviors. Thus, while the parasite's ejected nestling indeed has nothing to gain from retaliation, the parasite's future nestlings can benefit from this behavior, providing an explanation for the retaliation observed in nature^12^,^13^.

This explanation may appear surprising: for retaliatory behavior to be favored, hosts need to have evolved a plastic response. On the other hand, the ability to perform such a plastic behavior is only favored if already a large fraction of parasites retaliate. So how can both behaviors emerge in the first place? In our model, the host's ability to react conditionally comes without exogenous costs and benefits. In nature, however, the ability to react conditionally may have several additional advantages (e.g. a better defense against nest predators), but may also come with additional costs (e.g., as hosts need to process surrounding environmental cues). Ultimately the emergence of conditional behavior depends on an environment where it is beneficial for hosts to build up the capacity to react. As our model suggests, once hosts have acquired the ability to react conditionally, parasites can use clutch depredation as a way to enforce acceptance.

In experimental studies, retaliatory behavior is hard to distinguish from so called farming^1^,^13^,^22^, since they both involve depredation as a tactic. Whereas mafia parasites use depredation as a response to rejection, farming parasites depredate mature clutches before parasitizing them to synchronize the timing with the host, but not to induce a specific response. Both create opportunities for the parasite by forcing the host to re-clutch. In addition to depredation, retaliators monitor the host and ensure acceptance of their egg. As our primary interest was in the emergence of mafia behavior and conditional responses, we have refrained from including farming behavior (this would require a different type of model, that needs to track the age of the clutch). However, it is important to note that incorporating farming behavior cannot explain host acceptance. Only under retaliation can conditional acceptance of the host become beneficial.

In a previous model, the frequency of accepting hosts depended on the success of a second clutch^20^. In such a case, the parasite must ‘reward' the accepter hosts by not parasitizing the second clutch. But observations show that magpies lay only one clutch during a breeding season unless the parasite retaliated^22^. Our results show that accepter hosts will evolve as a response to retaliation and there is no need for the parasite to reward accepters, which is in line with experimental observations^1^,^13^,^22^. As accepter host frequency increases, it is no longer necessary for parasites to retaliate. Thus, non-retaliators can emerge and increase in frequency. Our model also suggests that if retaliation costs are high (eg. reducing total eggs laid, small *β_M_*) then the probability that a host is parasitised decreases. As a result hosts are more inclined towards rejection and thus parasites must retaliate to induce acceptance. Such a situation provides a glimpse into the frequency-dependence effects of parasitism probability. Hosts will accept if both the frequency of retaliatory parasites and the probability of parasitism are high. These results corroborate with a previous model^19^, however, we also show that the co-evolutionary arms race results in cycles with one species lagging behind the other, as shown in Fig. 2.

Our results are in line with observed patterns, suggesting that hosts learn^3^,^35^,^36^ and change their behavior as a response to mafia parasites within one breading season^22^. It is such learnt behavior from a repeated interaction that promotes the evolution of retaliation in our model. Typically, repeated interactions and co-evolutionary changes are thought to induce parasites to specialize on only one or a few hosts^37^. However, our results present retaliation as a behavior that the parasite can use to evade specialization and induce acceptance by the host. Other studies which consider manipulative behavior in parasitic species^21^,^38^,^39^ have suggested a similar effect of plasticity^40^,^41^. A general behavior, such as retaliation, forces a host to change it's response and as a result the parasite has a mechanism to manipulate multiple hosts without the need to specialise. We further speculate that the success of other parasitic species, which can infect multiple hosts, may be explained by the same mechanism.

## Methods

We simulated the evolutionary dynamics of this host-parasite interaction by assuming two finite populations of size *N_h_* and *N_p_* for hosts and parasites, respectively. Our model is based on constant population sizes, which is a good approximation if neither hosts nor parasites undergo large fluctuations in their abundance. Relaxing this assumption^25^,^42^,^43^ can lead to a different kind of dynamics which is not considered here. In each season a parasite will interact with multiple hosts. All parasites play *G* games repeatedly against randomly selected hosts, resulting in the game payoffs given in Fig. 3. In the beginning of each season, the host lays *b_h_* eggs in a clutch, which may become parasitized. The parasite's behavior is determined by the number of eggs laid per host nest *b_p_* (with *b_p_* < *b_h_*), whether it is a retaliator or not, and whether it returns to the same host or not. Thus, there are 4(*b_h_* − 1) possible strategies for parasites. The host's behavior is determined by the degree of parasitism it can detect or tolerate, *t* (with *t* < *b_h_*), whether mafia parasites are accepted after depredation or not, whether returning parasites are accepted or not, and whether the host ejects or abandons the entire nest, Fig. 3. For instance, depending on the host's threshold trait *t*, the host may accept the parasitism (if *b_p_* ≤ *t*) incurring a parasitism cost *c_p_* and a nestling cost *c_n_*; the parasite gains *b_p_* eggs. On the other hand, the host can reject the parasitism (if *b_p_* > *t*) either by ejecting the eggs or abandoning the nest, either way risking retaliation and re-parasitism by the parasite. In the case where these hosts avoid both retaliation and re-parasitism, ejector hosts incur *c_p_* while abandoner hosts incur a re-clutching cost, *c_r_*. Once rejected the parasite can decide to return, either for retaliation or parasitism, and the host can again decide to accept or reject (we assume only ejection in the third stage, as unconditional abandoners always have fitness of zero). Overall, this leads to 8*b_h_* possible strategies for the host. We assume non-overlapping generations in which individuals reproduce and contribute offspring to the next generation in proportion to their fitness while maintaining constant population sizes for both hosts and parasites (this corresponds to a Wright-Fisher process in population genetics). Offspring inherit the strategy of the parent. However, at each reproductive step mutations can occur with probability *μ_p_* for the parasite population and *μ_h_* for the host population, leading to a random phenotype. In comparison to the minimalistic model, where it was not necessary to include mutations, this time the strategies are coded by a collection of discrete “genes” that can mutate independently, which produces natural transitions from one strategy to the next.

## Author Contributions

M.A.C., C.H. and A.T. were equally involved in the design and analysis of the model and all authors wrote the paper.

## References

[b1] Davies N. B. & Quinn D. Cuckoos, Cowbirds and Other Cheats (T and A D Poyser, London, 2000).

[b2] Krüger O. Cuckoos, cowbirds and hosts: adaptations, trade-offs and constraints. Philos. Trans. R. Soc. B. 362, 1873–1886 (2007). 10.1098/rstb.2006.1849 PMC2442387 17827098

[b3] Davies N. B. & Welbergen J. A. Social transmission of a host defense against cuckoo parasitism. Science 324, 1318–1320 (2009). 19498167 10.1126/science.1172227

[b4] Davies N. B. Cuckoo adaptations: trickery and tuning. J Zool. 284, 1–14 (2011).

[b5] Lotem A. Learning to Recognize Nestlings Is Maladaptive for Cuckoo Cuculus-Canorus Hosts. Nature 362, 743–745 (1993).

[b6] Brooke M. D. & Davies N. B. Egg Mimicry by Cuckoos Cuculus-Canorus in Relation to Discrimination by Hosts. Nature 335, 630–632 (1988).

[b7] Davies N. B. & Brooke M. D. Cuckoos Versus Reed Warblers - Adaptations and Counter-adaptations. Anim. Behav. 36, 262–284 (1988).

[b8] Brooker M. & Brooker L. Acceptance by the splendid fairy-wren of parasitism by Horsfield's bronze-cuckoo: Further evidence for evolutionary equilibrium in brood parasitism. Behav. Ecol. 7, 395–407 (1996).

[b9] Brooker L. & Brooker M. Why do splendid fairy-wrens always accept cuckoo eggs? Behav. Ecol. 9, 420–424 (1998).

[b10] Winfree R. Cuckoos, cowbirds and the persistence of brood parasitism. Trends Ecol. Evol. 14, 338–343 (1999). 10441306 10.1016/s0169-5347(99)01643-2

[b11] Zahavi A. Parasitism and Nest Predation in Parasitic Cuckoos. Am. Nat. 113, 157–159 (1979).

[b12] Soler M., Soler J. J., Martinez J. G. & Møller A. P. Magpie host manipulation by great spotted cuckoos: evidence for an avian mafia? Evolution 49, 770–775 (1995). 10.1111/j.1558-5646.1995.tb02312.x 28565143

[b13] Hoover J. P. & Robinson S. K. Retaliatory mafia behavior by a parasitic cowbird favors host acceptance of parasitic eggs. Proc. Natl. Acad. Sci. USA 104, 4479–4483 (2007). 17360549 10.1073/pnas.0609710104 PMC1838626

[b14] May R. M. & Robinson S. K. Population-Dynamics of Avian Brood Parasitism. Am. Nat. 126, 475–494 (1985).

[b15] Lawes M. J. & Marthews T. R. When will rejection of parasite nestlings by hosts of nonevicting avian brood parasites be favored? A misimprinting-equilibrium model. Behav. Ecol. 14, 757–770 (2003).

[b16] Lotem A., Nakamura H. & Zahavi A. Rejection of Cuckoo Eggs in Relation to Host Age - a Possible Evolutionary Equilibrium. Behav. Ecol. 3, 128–132 (1992).

[b17] Harrison M. D. & Broom M. A game-theoretic model of interspecific brood parasitism with sequential decisions. J. Theor. Biol. 256, 504–517 (2009). 18977367 10.1016/j.jtbi.2008.08.033

[b18] Svennungsen T. O. & Holen Ø. H. Avian Brood Parasitism: Information Use and Variation in Egg-Rejection Behavior. Evolution 64, 1459–1469 (2010). 20015240 10.1111/j.1558-5646.2009.00919.x

[b19] Robert M. *et al.* Retaliatory cuckoos and the evolution of host resistance to brood parasites. Anim. Behavi. 58, 817–824 (1999). 10.1006/anbe.1999.1205 10512655

[b20] Pagel M., Møller A. P. & Pomiankowski A. Reduced parasitism by retaliatory cuckoos selects for hosts that rear cuckoo nestlings. Behav. Ecol. 9, 566–572 (1998).

[b21] Soler J. J., Møller A. P. & Soler M. Mafia behaviour and the evolution of facultative virulence. J. Theor. Biol. 191, 267–277 (1998). 9631567 10.1006/jtbi.1997.0599

[b22] Soler J. J., Sorci G., Soler M. & Møller A. P. Change in host rejection behavior mediated by the predatory behavior of its brood parasite. Behav. Ecol. 10, 275–280 (1999).

[b23] Brosnan S. F., Salwiczek L. & Bshary R. The interplay of cognition and cooperation. Philos. Trans. R. Soc. B 365, 2699–2710 (2010). 10.1098/rstb.2010.0154 PMC2936177 20679113

[b24] Hofbauer J. & Sigmund K. Evolutionary Games and Population Dynamics (Cambridge University Press, Cambridge, 1998).

[b25] Broom M. & Rychtář J. Game-Theoretical Models in Biology (Chapman and Hall/CRC, 2013).

[b26] Krüger O. Brood parasitism selects for no defence in a cuckoo host. Proc. R. Soc. B 278, 2777–2783 (2011). 10.1098/rspb.2010.2629 PMC3145182 21288944

[b27] Guilford T. & Read A. F. Zahavian Cuckoos and the Evolution of Nestling Discrimination by Hosts. Anim. Behav. 39, 600–601 (1990).

[b28] Goeree J. K. & Holt C. A. Ten little treasures of game theory and ten intuitive contradictions. Am. Econ. Rev. 91, 1402–1422 (2001).

[b29] Clutton-Brock T. H. & Parker G. A. Punishment in animal societies. Nature 373, 209–216 (1995). 7816134 10.1038/373209a0

[b30] Fehr E. & Gächter S. Altruistic punishment in humans. Nature 415, 137–140 (2002). 11805825 10.1038/415137a

[b31] Herrmann B., Thöni C. & Gächter S. Antisocial punishment across societies. Science 319, 1362–1367 (2008). 18323447 10.1126/science.1153808

[b32] Dreber A., Rand D. G., Fudenberg D. & Nowak M. A. Winners don't punish. Nature 452, 348–351 (2008). 18354481 10.1038/nature06723 PMC2292414

[b33] Traulsen A., Röhl T. & Milinski M. An economic experiment reveals that humans prefer pool punishment to maintain the commons. Proc. R. Soc. B 279, 3716–3721 (2012). 10.1098/rspb.2012.0937 PMC3415907 22764167

[b34] Hilbe C. & Traulsen A. Emergence of responsible sanctions without second order free riders, antisocial punishment or spite. Sci. Rep. 2, 458 (2012). 22701161 10.1038/srep00458 PMC3374160

[b35] Griffin A. S. Social learning about predators: a review and prospectus. Learn. Behav. 32, 131–140 (2004). 15161148 10.3758/bf03196014

[b36] Ishii Y. & Shimada M. Learning predator promotes coexistence of prey species in host-parasitoid systems. Proc. R. Soc. B 109, 5116–5120 (2012). 10.1073/pnas.1115133109 PMC3324012 22411808

[b37] Rothstein S. I. Relic behaviours, coevolution and the retention versus loss of host defences after episodes of avian brood parasitism. Anim. Behav. 61, 95–107 (2001). 11170700 10.1006/anbe.2000.1570

[b38] Ponton F. *et al.* Water-seeking behavior in worm-infected crickets and reversibility of parasitic manipulation. Behav. Ecol. 22, 392–400 (2011). 22476265 10.1093/beheco/arq215 PMC3071748

[b39] González-Forero M. & Gavrilets S. Evolution of Manipulated Behavior. Am. Nat. 184, 439–451 (2013). 10.1086/671932 24021397

[b40] Taylor C. & Nowak M. A. Evolutionary game dynamics with non-uniform interaction rates. Theor. Pop. Biol. 69, 243–252 (2006). 16427669 10.1016/j.tpb.2005.06.009 PMC2880897

[b41] Restif O. An offer you cannot refuse: down regulation of immunity in response to a pathogen's retaliation threat. J. Evol. Biol. 26, 2021–2030 (2013). 23927686 10.1111/jeb.12209 PMC4274018

[b42] Argasinski K. Dynamic multipopulation and density dependent evolutionary games related to replicator dynamics. A metasimplex concept. Math. Biosci. 202, 88–114 (2006). 16797041 10.1016/j.mbs.2006.04.007

[b43] Gokhale C. S., Papkou A., Traulsen A. & Schulenburg H. Lotka-Volterra dynamics kills the Red Queen: population size fluctuations and associated stochasticity dramatically change host-parasite coevolution. BMC Evol. Biol. 13, 254 (2013). 24252104 10.1186/1471-2148-13-254 PMC4225518

